# Identifying Critical Nutrient Intake in Groups at Risk of Poverty in Europe: The CHANCE Project Approach

**DOI:** 10.3390/nu6041374

**Published:** 2014-04-02

**Authors:** Marina Nikolić, Maria Glibetić, Mirjana Gurinović, Jelena Milešević, Santosh Khokhar, Stefania Chillo, Jonas Algis Abaravicius, Alessandra Bordoni, Francesco Capozzi

**Affiliations:** 1 Institute for Medical Research, Centre of Research Excellence in Nutrition and Metabolism, University of Belgrade, Tadeuša Košćuška 1, Belgrade 11000, Serbia; E-Mails: marina.nikolic12@yahoo.com (M.N.); mirjana.gurinovic@gmail.com (M.G.); jelena.milesevic@gmail.com (J.M.); 2 School of Food Science and Nutrition, University of Leeds, Leeds LS2 9JT, UK; E-Mails: chance@unibo.it (S.K.); s.chillo@hotmail.com (S.C.); 3 Faculty of Medicine, Department of Physiology, Biochemistry, Microbiology and Laboratory Medicine, Vilnius University, Vilnius LT-03101, Lithuania; E-Mail: algis.abaravicius@mf.vu.lt; 4 Department of Agriculture and Food Science and Technology, University of Bologna, Piazza Goidanich 60, Cesena 47023, Italy; E-Mails: alessandra.bordoni@unibo.it (A.B.); francesco.capozzi@unibo.it (F.C.)

**Keywords:** poverty, critical nutrients intake, Europe, CHANCE food

## Abstract

The aim of the CHANCE project is to develop novel and affordable nutritious foods to optimize the diet and reduce the risk of diet-related diseases among groups at risk of poverty (ROP). This paper describes the methodology used in the two initial steps to accomplish the project’s objective as follows: 1. a literature review of existing data and 2. an identification of ROP groups with which to design and perform the CHANCE nutritional survey, which will supply new data that is useful for formulating the new CHANCE food. Based on the literature review, a low intake of fruit and vegetables, whole grain products, fish, energy, fiber, vitamins B1, B2, B3, B6, B12 and C, folate, calcium, magnesium, iron, potassium and zinc and a high intake of starchy foods, processed meat and sodium were apparent. However, the available data appeared fragmented because of the different methodologies used in the studies. A more global vision of the main nutritional problems that are present among low-income people in Europe is needed, and the first step to achieve this goal is the use of common criteria to define the risk of poverty. The scoring system described here represents novel criteria for defining at-risk-of-poverty groups not only in the CHANCE-participating countries but also all over Europe.

## 1. Introduction

It has long been recognized that food patterns, nutrient intakes and health outcomes vary according to indicators of socio-economic conditions [1], such as income. Low-income people are reportedly less likely to consume a healthy diet than wealthier people, and energy-dense/nutrient-poor diets are preferentially consumed by persons of lower socioeconomic status [2,3]. Consequently, their health is at greater risk from diet-related illness.

A diverse range of natural (e.g., fruits and vegetables, whole grains) or enriched/fortified foods with a higher health value, which would be beneficial to health and are offered at European markets, are too expensive for those earning a low income. In response, international health authorities encourage governments at the national level to improve the diets of low-income people in a sustainable way, that is, to make healthy food affordable for all [3,4]. However, limited efforts are currently directed at developing healthier products targeted at a lower price range.

In aiming to address barriers to the consumption of a healthy diet in at risk of poverty (ROP) groups, the European Commission supported the launch of the CHANCE project. The full title of the project clarifies its objective: a multidisciplinary group of nutritionists, food chemists, economists and technologists will use “Low cost technologies and traditional ingredients for the production of affordable, nutritionally correct foods for improving health in population groups at risk of poverty”. Specifically, in addition to its low cost, a fundamental property of novel CHANCE foods will be an optimal concentration of the nutrients that are most critical to the health of low-income adults and the elderly in Europe. The overall strategy of the CHANCE project is the development and exploitation of a nutritional approach based on affordable, nutritionally correct, tasty food products, which, together with healthy lifestyle changes, will reduce the impact of low incomes on health status. The objectives will be pursued by applying the following integrated key actions: (1) identification of critical nutritional intakes, *i.e.*, nutritional deficiencies and over-nutrition, and barriers towards healthier eating, in representative individuals of specific groups at ROP; (2) selection of ingredients and raw materials to be exploited for the formulation of some new CHANCE food prototypes on the basis of low-cost ingredients, and processing and packaging technologies to target the identified critical intakes.

Two different approaches can be used to identify critical nutritional intake and barriers to healthier eating as follows: (a) the review of existing data, and (b) the production of new data. Accordingly, the first step within the CHANCE project was to review the existing literature concerning nutritional habits in low-income populations. Although it is generally accepted that earning a low income has a negative impact on the quality of the diet, the existing information on nutritional quality and possible malnutrition in economically disadvantaged populations is highly fragmented. The data in the literature come from studies that have used different methodologies to assess food consumption and to define the quality of the diet. In addition, the definition of “poverty” is often based on different parameters.

Therefore, in addition to the existing data in the literature, it was agreed that there was a need to create a more global vision of the primary nutritional problems that are present in low-income people in Europe. This common vision can only be achieved by using common criteria to define the risk of poverty, and a common methodology to investigate the dietary habits of low-income people in different countries. This way, it is possible to identify the nutrients for which intake is critical in almost all countries and subgroups of the population; these critical intakes represent the basis for the production of low-cost, nutritionally valued food that is intended for low-income people all over Europe.

In addition to the methodology used to perform the literature review and the primary findings related to this activity, this paper describes the methodological approach used to uncover the primary nutritional problems in low-income subjects from the five countries involved in the CHANCE project (Finland, Italy, Lithuania, Serbia and the United Kingdom).

Representative at risk of poverty (ROP) groups were selected by using a harmonized definition of poverty to enable cross-country comparison (Step 1). They were used as a target population to investigate the dietary habits of low-income people in different countries by using the same methodology (Step 2). This makes it possible to identify the nutrients for which the intake is critical in almost all countries and subgroups of the population and to integrate the results with data in the literature and with information on perceived obstacles to healthy eating (skills for cooking healthfully, knowledge of healthy eating, *etc*.) obtained in parallel surveys by CHANCE co-workers (Step 3).

Overall, this integrated approach allows for the design of new affordable and nutritionally correct foods intended to improve the diet of at-risk-of poverty adults and the elderly in Europe (Step 4).

**Figure 1:**
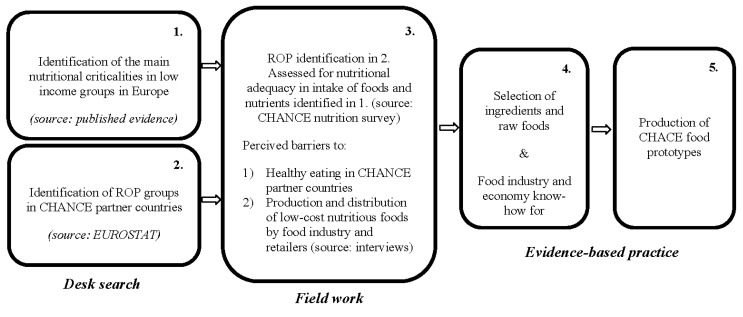
The CHANCE strategy for developing new and affordable nutritionally correct foods and meals in order to improve the diet of population groups at risk of poverty (ROP).

The overall strategy of the CHANCE project is shown in Figure 1.

## 2. Methods

### 2.1. Methodology for the Literature Review

A search of studies on the dietary intake of foods and/or nutrients in low-income groups in Europe was conducted in electronic literature databases (Medline and Web of Science). Suitable grey literature reports, primarily those issued by the food and nutrition authorities the World Health Organization (WHO), the Food and Agriculture Organization (FAO), the European Food Safety Authority (EFSA) and those resulting from European Commission (EC)-funded projects (EURRECA, EPIC, EFCOVAL) were used for discussion only.

Schematically, the Medline and Web of Science database search was built by using the following combination of concepts: [socioeconomic status (income, low-income, poverty, inequality, living standard, social class, socio-economic status, education, employment, occupation; and their combinations)] AND [dietary intake (diet, eating, consumption, food habits, food preferences, requirement, recommended, guideline, adequacy, inadequacy, deficiency, nutritive value, nutrition assessment, energy intake; and their combinations)] AND [foods/nutrients (food, food groups, food subgroups, nutrient, micronutrient, macronutrient, vitamin, mineral, cereal, bread, starch, fiber, carbohydrate, protein, fat, fruit, vegetable, milk, milk products, dairy products, cheese, meat, meat products, fish, oil, sugar, sweet, cake, pastry, nuts, seeds, fast food, processed food; and their combinations)] NOT [clinical trials OR patients]. For each concept, the database-specific indexing terms (MeSH or Web of Science terms) were searched in addition to terms in the title or abstract. Potentially relevant documents resulting from EC-funded projects were gathered through their websites and by contacting the project coordinators. An additional manual search was performed by checking the reference lists of the key publications from the health authorities (WHO, FAO, and EFSA). The output tables captured key characteristics of the following: (a) study methodologies, that is, the country and year of the study, the number of subjects and their genders, the sample (age range), foods and/or nutrients under study, dietary assessment method, income classification; (b) primary (qualitative) study outcomes, that is, whether estimated food and/or nutrient intakes were in accordance with recommended levels or not, in addition to findings on the observed differences in food and/or nutrient intakes between low- and high-income groups, if any.

We only considered full-text articles on studies conducted between 1 January 1990 and 31 July 2013 among apparently healthy low-income adults and/or the elderly in Europe (*i.e.*, studies examining disease or patient subgroups or institutionalized groups were excluded) that were published in English. The search was limited to adults and/or the elderly because children were not considered as part of the target group, as explained in the following paragraph. The studies had to be cohorts, case-control or cross-sectional studies that provided information on the self-reported dietary intakes of individual foods and/or nutrients, the levels of which were compared within the study (a) to food-based dietary guidelines and/or dietary reference values, respectively; and/or (b) between low-income and high-income groups. The latter were included because a body of evidence suggests better compliance with dietary recommendations and guidelines among affluent groups than among those that are economically disadvantaged [3,5].

### 2.2. Identification of ROP Groups for the CHANCE Survey

To identify ROP groups in the CHANCE countries, the definition of poverty, the “at-risk-of-poverty threshold”, and the data on “income distribution and monetary poverty” from EUROSTAT [6] were used. EUROSTAT [6] is the official statistical institution of the European Union, and it is responsible for providing the European Union with statistics on income, social inclusion and living conditions at the European level. The primary role of EUROSTAT is to define common methodology and to process and publish comparable statistical information at the European level. The EUROSTAT definition of ROP is officially accepted by the European Community, providing an advantage of a standard that makes the data comparable within European Countries.

Accordingly, the risk of poverty is defined as “the share of people with an equivalised disposable income (after social transfer) below the at-risk-of-poverty threshold, which is set at 60% of the national median equivalized disposable income”. The threshold calculation is based on the weights used, for example, by the Organization for Economic Co-operation and Development [7] in equivalizing household income to consider the number of people in a household. These weights are as follows: One for the first adult in the household; 0.5 for each additional adult in the household; and 0.3 for each child in the household. All people under 14 years of age are considered to be children, and people 14 years or older are adults. Thus, income thresholds were calculated using the following Formula 1:

60% threshold = 0.6 × MDN × (1 + 0.5 *a* + 0.3 *b*) (1)

in which 0.6 = ROP-threshold; MDN = annual median income; 1= the weight of the first adult of the household; *a* = the number of people above 14 years of age in the household; and *b* = the number of people below 14 years of age in the household. For example, the national equivalized household median income in Italy (according to EUROSTAT) is approximately 15,640. The upper threshold for a household with two adults and two children is then 0.6 × 15640 × (1 + 1 × 0.5 + 2 × 0.3) = 19706.40 euro/year.

Information on the percentage and absolute number of ROP in groups stratified by country and age (young adults 18–24 year, adults 25–64 year, and elderly ≥65 year) were extracted from the EUROSTAT database; an alternative source that applied EUROSTAT methodology was used for Serbia [8].

The ROP groups with the highest percentage and absolute sizes were identified according to their age and gender in each country. This led to a higher homogeneity of the investigated ROP population in each country and a representation of the group with the highest risk of poverty within each nation.

Children were not assumed as a target group. Assessing dietary intake in people of any age is challenging but measuring the diet of infants and children can be particularly problematic. Young children may lack the cognitive skills, writing skills and food knowledge to record their own food intake. Multiple people may be responsible for the care of the child and the collection of an accurate picture of intake may necessitate the combination of parental reports with observations in school or the nursery. Where interviews are conducted with the child themselves, questions may need to focus on aspects of the diet to which children are likely to may attention. For example, children may not be familiar with food names or brands but may be able to describe their texture, color and images on packaging [9] Furthermore, a child’s diet is highly controlled by older members of the household, and children do not have a reliable opinion with regards to most of the questions in the survey.

A selection of the most representative ROP group in each country was calculated as follows: each proportion and absolute number of ROP within the defined age range and country were assigned values from one to three from lowest to highest, and the assigned values were subsequently multiplied to obtain the total CHANCE score. The group that scored the highest was chosen from each country. For example, the following proportions (%) and absolute numbers (thousands people) of ROP within relevant age ranges were reported in Finland—26.5 (116), 10.7 (306) and 18.9 (174) for 18–24, 25–64 and ≥65 years, respectively, so the corresponding CHANCE total scores were 3 × 1 = 3, 1 × 3 = 3 and 2 × 2 = 4. Clearly, the subgroup aged >65 years scored the highest and was hence chosen as a target ROP group from Finland. Choices of other country-specific ROP groups were based on analogous calculations.

## 3. Results

### 3.1. Nutritional Data Retrieved from the Literature

**Figure 2:**
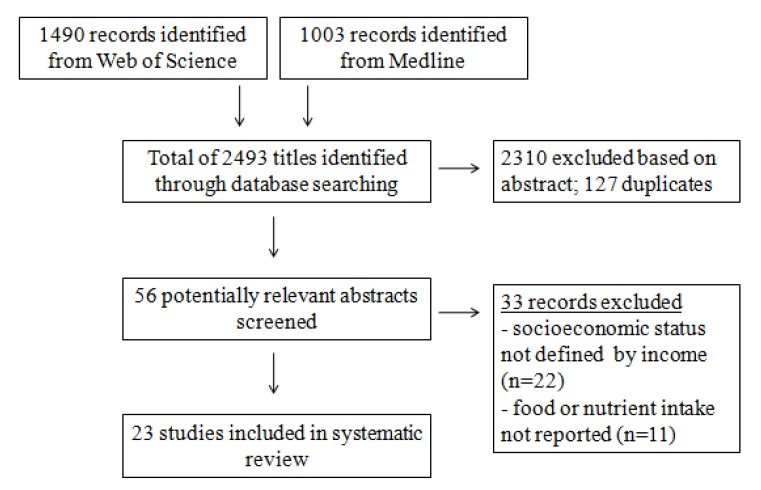
Flow diagram of literature search and study selection.

The retrieval of relevant studies from Medline and Web of Science databases was part of a wider search process to identify studies on nutritional criticalities among socioeconomically disadvantaged groups in Europe. The wider searches involved search terms for foods and/or nutrients as follows: macronutrients, vitamins, minerals, and a list of food groups based on the ones in the EPIC project [10], and search terms that reflect socioeconomic status, *i.e.*, income, education and occupation. For the purpose of the current paper, only studies that reported the dietary intake of foods and/or nutrients by income were included (Figure 2).

Sample characteristics and poverty indicators are shown in Table 1. We identified 23 studies that complied with inclusion criteria, out of which 17 were cross-sectional and six were prospective cohorts, with sample sizes that ranged from 100 [11] to 69,383 [12].

**Table 1:** Characteristics of included studies.

Country and Year	Number of Subjects and Gender	Sample (Age Range)	Dietary Assessment Method	Classification of Income
AUT 2006 [13]	261 F	Pregnancy (<45)	24 HDR	Low household income: <1090 EUR; Middle household income: 1090–2180 EUR; High household income >2180 EUR
BGR 2002 [14]	2133 M & F	Random sample (all ages)	FFQ	Amount of household income (continuous variable)
BEL 2007 [11]	50 M, 50 F	Low-income population (>18)	FFQ and 24 HDR	Living in the average family: monthly income of max 400 €/person/month; Living alone: monthly income of max 700 €/month
CZE, FIN, POL, RUS 2010 [15]	8429 M, 12,897 F	Random sample (40–69)	FFQ	Four categories of economic difficulty: frequently, occasionally, rarely and never
DNK 2001 [16]	852 M, 870 F	Random sample(20–67)	7-day estimated dietary record	Five categories depending on the amount of income in DKK
FIN 1996 [17]	870 M, 991 F	Random (25–64)	3-day non-weighed dietary record	Family (household) income quartiles
FIN 2003 [18]	9324 M, 10,658 F	Random sample (25–64)	Food behavior survey	Income quintiles
FIN 2008 [12]	33,302 M, 36,081 F	Random sample (25–64)	FFQ	Income quintiles
FIN 2010 [14]	1792 M, 7168 F	Random sample (40–60)	FFQ	Income quartiles
FRA 2010 [19]	133 M, 162 F	Random sample (30–60)	FFQ	Financial situation of the household (It is satisfactory, or we need to pay attention; We barely manage, or It is hard not to go into debt)
GRC 2004 [20]	1933 F	Random sample of Greek and Albanian mothers (24–31)	30-day food diary	Three categories by income per month (high, moderate, and low)
ITA 2012 [21]	133 M, 173 F	Random sample (>65)	FFQ	Two categories by self-estimated household income as sufficient or insufficient
POL 2004 [22]	1001 M, 836 F	Random sample (18–64)	FFQ	5 categories based on the amount of income per month per person (<75 EUR; 75–124 EUR; 125–249 EUR; 250–374 EUR; >374 EUR)
POL 2007 [11]	215 M & F	Low-income population (>18)	FFQ and 24 HDR	The income ceiling was set at 1100 PLN for a single person and 800 PLN per person for respondents living in the household
PRT 2004 [23]	18,663 M, 20,977 F	Random sample (>18)	FFQ	4 categories based on the amount of income per month per person (<314 EUR; 315–547 EUR; 548–815 EUR; >815 EUR)
SWE 2006 [24]	57,000 M & F	Random sample (18–84)	FFQ	Two categories based on the amount of income per person
UK 1997 [5]	7000 M & F	Random sample (all age groups)	Household food consumption survey	SEC: A-the highest income group and D & E2-the low income groups
UK 1998 [25]	177 M, 192 F	Random sample (>65)	FFQ and 4 days dietary diaries	Respondents were asked to identify in which range of income levels they were; income was then calculated as a proportion of the basic state pension, taking into account whether respondents were living alone or as a couple
UK 1999 [26]	284 M & F	Random sample (>65)	4 days weighed dietary record	Low income: <£6000 per annum and High income: >£6000 per annum
UK 2003 [27]	150 M, 530 F	Low-income population (17–100)	Food behavior survey	Low-income housing association
UK 2004 [28]	1724 M & F	Random sample (19–64)	7 days WFR	Receiving and not receiving state benefits
UK 2007 [29]	3728 M & F	Low income (All age groups)	4 × 24 HDR	A doorstep screening questionnaire was used to establish eligibility for inclusion in the survey, based on markers of deprivation
UK 2009 [30]	1461 F	Pregnancy (mean age = 30.4)	FFQ	Scottish Index of Multiple Deprivation (5 categories)
UK 2009 [31]	11,044 M & F	Random adults (>18)	FFQ	Household income data are reported in the data set

Abbreviations: AUT—Austria; BGR—Bulgaria; BEL—Belgium; CZE—Czech Republic; FIN—Finland; POL-Poland; RUS—Russia; DNK—Denmark; FRA—France; GRC—Greece; ITA—Italy; PRT—Portugal; SWE—Sweden; UK—United Kingdom; F—females; M—males.

Foods and/or nutrient intakes were the examined dietary outcomes: twelve studies examined the intake of foods, two studies examined the intake of nutrients, and nine studies reported the estimated intake of both foods and nutrients. All studies compared estimates of food and/or nutrient intake between low- and high-income groups, with the exception of three studies [11,27,29] in which the appropriate scientific methods were used to compare dietary reference values with food-based dietary guidelines. In two studies [17,28], food intakes were compared between income groups and nutrient intakes were compared with reference values and between income groups. Diverse dietary assessment methods were used to estimate the food and/or nutrient intakes: a 24-h diet recall (24HDR) [11,13,29], food frequency questionnaire (FFQ) [11,12,14,15,19,21,22,23,24,25,30,31,32], diet record (DR) [16,17,20,25,26,28], food behavior surveys [18,28], and household food consumption survey [5] were employed, whereas an FFQ along with 24 HDR and 4 days of DR were used in two studies [11,25].

These studies applied multiple approaches to define low-income groups as follows: the amount of household income as a continuous variable [14,25,31], the personal income categorized into five [12,16,18,22,30,32], four [15,17,23], three [13,20] and two categories [5,19,21,24,26,28]. Three studies only analyzed the low-income populations that were identified on the basis of deprivation markers [29] and income threshold [11], or by low-income housing associations [27].

Qualitative findings on food intake among low-income groups are summarized in Table 2. The data were most abundant for the intake of fruit and/or vegetables (19 studies), dairy products (11 studies), cereals, cereal products and starchy foods (including potatoes) (eight studies), and meat and/or processed meat (nine studies).

**Table 2:** A summary of reported findings on food intake among low-income populations from the included studies.

Food Groups	Intake Lower than in High-Income Groups/Recommended *	Intake Higher than in High-Income Groups/Recommended *	No Differences
Fruits and vegetables	[11,27,29] *, [19,24,28,30,31,32], ([16], F), [5,26], fruit ([17], M), ([15], FIN), [25], vegetables [12,17,18]	[14]	[21], ([16], M) fruit ([17], F)
Dairy products	[14,21], low fat milk ([15], FIN), cheese ([17], F), butter [11] ^¥^^,^*, milk ([23], M)	milk [11] ^§^^,^*, ([17], F), [30], butter [17], full cream milk [29] *, [5], cream [30]	cheese & skimmed milk [16], cheese ([27], M), milk ([17], M), ([23], F])
Cereals and grains	whole grain bread [11] *, [29] *, [5,15]	[5], starchy foods [11] ^§^^,^*, ([23], M), white bread [11] ^¥^^,^*, [17]	[21], starchy foods [17], ([23], F)
Potatoes	-	[5], [11] ^¥^^,^*, ([17], F), fried potatoes [30]	([17], M)
Meat	[11] ^¥^^,^*, [14], ([23], M), [30], red meat [21], poultry meat [11] ^§^^,^*	[29] *, ([17], F), pork & beef meat [11] ^§^ *	([17], M), ([23], F), poultry meat [21]
Meat products and processed meat	-	[29] *, [5,30]	[17]
Fish	[11,29] *, ([17], F), [21,30]	-	([17], M), [16]
Fat	olive oil [11] *, vegetable fat spread ([15], FIN & POL), recommended cooking spreads ([15], FIN-F & RUS-M), margarine [11] ^¥^^,^*	[29] *, animal fats [14], margarine [11] ^§^^,^*	recommended cooking spreads ([15], FIN-M & RUS-F), margarine [17], vegetable oil [22]
Cakes, biscuits, sugar and confectionary products	-	[5,13 *,^26^], desserts ([17], F)	desserts [17], M
Soft drinks	[17], M	[29] *, [30]	[17], F
Alcoholic beverages	([22], F), [30]	[24]	[17,28], ([22], M), [18]
Soup, bouillons	-	[17], M	[17], F

* studies that compared food intakes with dietary recommendations; ^§^ Belgium; ^¥^ Poland; F—females; M—males; FIN—Finland; POL—Poland; RUS—Russia.

Only one study [14] reported a higher intakes of fruit and/or vegetables among low-income than among high-income groups. One study showed no differences in the fruit and vegetable intake by income [21], whereas 17 other studies found the opposite. Three studies [11,27,29] showed that the consumption of fruits and vegetables among low-income groups did not comply with dietary recommendations. There were inconsistent results for the differences between low- and high-income groups in dairy product intake. Only two studies compared the intake of dairy products with food-based dietary guidelines, the observed intakes were above the recommendations [11,29]. The intake of whole grain bread [5,15] was lower in low-income than in high-income groups; additionally, the intake of cereals, cereal products and starchy foods (including potatoes) were generally higher in low-income than in high-income groups [5,17,23,30]. One study compared the intake of cereals and cereal products among low-income groups with food-based guidelines as follows: Whole grain cereals were consumed less, whereas white bread, starchy food and potatoes were consumed more often than recommended [11]. The intake of fish was below the recommended amounts [11,29] and lower among low-income than among high-income females [17,21,30], whereas one study showed no differences in fish intake by income [16]. Processed meat was consumed more among low-income than among high-income groups [5,30], although one study found no differences [17]. Findings for meat intake by income were inconsistent; one study showed that meat intake by low-income groups was below the recommendation [11]. Three studies reported inconsistent findings for differences in fat consumption between income groups [15,17,22]; one study found a lower intake of olive oil and a higher intake of vegetable fats among low-income populations than recommended [11]. Data on the intake of cakes, biscuits, sugar and confectionary products showed higher intakes in low-income than in high-income groups [5,17,30]. Five studies on the use of alcohol reported inconsistent results [17,18,22,24,28,30]. Findings on the consumption of soft drinks and bouillons were limited and inconsistent [17,29,30]. No studies were found on the intake of egg and egg products, nuts and seeds, and condiments and sauces.

The qualitative findings of nutrient intakes among low-income groups are summarized in Table 3. The data were most abundant for the intake of proteins (eight studies), energy, total fat, saturated fat, and vitamin A (six studies); carbohydrates, vitamin B2, iron, magnesium and potassium (five studies for each). 

The energy intake was reportedly below recommended levels [11,29] and lower in low-income than in high-income groups [26,28], whereas two studies found no differences in the energy intake by income [17,30]. Data on carbohydrate and protein intake were inconsistent. None of the six studies on fat intake reported low intakes; this nutrient was higher than recommended [11,17] and higher in low-income than in high-income groups [20], or no differences could be observed [11,16,17,28,30]. Mono-unsaturated fatty acids (MUFA) and poly-unsaturated fatty acids (PUFA) intakes were either lower than recommended [29] or no differences between income groups could be found [11,30]. Saturated fat intake results were inconsistent. The fiber intake was lower in low-income than in high-income groups [26,30], and it was lower than recommended [11,17].

Data on the intake of vitamin A and C were abundant (six and four studies, respectively), but the findings were inconsistent. Information on the intake of vitamins D and E was limited. Intakes of B-vitamins (B1, B2, B3, B6 and B12) were, in general, lower in low-income than in high-income groups [20,26,30], and below the recommended levels [11,28,29]. Two studies on folate intake showed lower intakes in low-income than in high-income groups [26,30], and one study showed no differences in folate intake by income [20].

Four studies were found for calcium; two studies indicated it was below the recommended levels [11,29] and lower in low-income then in high-income groups [26]; only one study found no differences between income groups [30]. Iron intake was lower in low-income than in high-income groups [26,30], whereas contrasting findings were observed when the intakes were compared with reference values [11,28,29]. Data on magnesium and potassium intake indicated that the reported levels were below reference values [11,28,29] and lower in low-income than in high-income groups [26,30]. Zinc intake was lower in low-income than in high-income groups [30] and lower than recommended [11,29]. Three studies [11,29,30] indicated that sodium intakes were higher than recommended, with the exception of a low-income population in Belgium [11]. Data on the intake of copper, iodine, manganese, phosphorus and selenium by income were scarce [11,29,30].

**Table 3:** Summary of reported findings on nutrient intake among low-income populations from the included studies.

Macronutrients	Intake Lower than in High-Income Groups/Recommended *	Intake Higher thanin High-Income Groups/Recommended *	No Differences
Energy	[11,29] *, [26,28]	-	[17,30]
Alcohol	[30], ([18], F)	-	[17,28], ([18], M)
Total carbohydrates	[11] ^¥,^*, [17] *, [26]	([17], F), [20]	[11] ^§^^,^*, ([17], M), [30]
Fiber	[11,17] *, [26,30]	-	[17]
Proteins	([28], F), [20,26,30]	[11] ^§^^,^*, [29] *, [13]	[11] ^¥^^,^*, ([28], M), [17]
Total fats	-	[11] ^§^^,^*, [17] *, [20]	[11] ^¥,^*, [16,^17^,^28^,^30^]
MUFA	[29] *	-	[11] *, [30]
PUFA	[29] *	-	[11] *, [30]
SFA	[29] *, [17]	[11] *, [18,20]	[17,30]
Vitamins			
Vitamin A	[28,29] *, ([17], M), [26,30]	-	[11] *, ([17], F), [30]
Vitamin B1	[11] ^¥,^*, [26]	-	[11] ^§,^*, [30]
Vitamin B2	[11,28,29] *, [26]	-	[30]
Vitamin B3	[11] ^¥,^*, [30]	-	-
Vitamin B6	[11] ^¥,^*, [30]	-	-
Vitamin B12	[20,26]	-	-
Vitamin C	[11] ^§,^*, ([17], M), [26,30]	-	[11] ^¥,^*, ([17], F), [17] *
Vitamin D	[29] *	-	[30]
Vitamin E	-	-	[11] ^¥,^*, [30]
Folate	[26,30]	-	[20]
**Minerals**			
Calcium	[11,29] *, [26]	-	[30]
Copper	[11] ^¥,^*, [30]	[11] ^§^ *	-
Iodine	[29] *	-	[30]
Iron	[11] ^¥,^*, [29] *, ([28] *, F), [26,30]	-	[11] ^§,^*, ([28] *, M)
Magnesium	[11,28,29] *, [26,30]	-	-
Manganese	[30]	-	-
Phosphorus	[30]	[11] *	-
Potassium	[11] ^¥,^*, [28,29] *, [26,30]	-	-
Selenium	[30]	-	-
Sodium	[11] ^§,^*	[11] ^¥,^*, [29] *, [26,30]	-
Zinc	[11] ^¥,^*, [29] *, [30]	[11] ^§,^*	-

* denotes studies that compared the nutrient intake with dietary recommendations; ^§^ Belgium; ^¥^ Poland; F—females; M—males.

### 3.2. Identifying the Most Representative ROP Groups in Participating Countries

The at-risk-of poverty rate (% of ROP, with thousands of people in parentheses) for the total population and by selected age groups (18–24, 25–64 and ≥65 years) and countries (Finland, Italy, Lithuania, Serbia and UK), and a calculation of the CHANCE total score are presented in Table 4.

The average percentage of the ROP in the total EU population was 16.9%, whereas it ranged from 13.7 in CHANCE partner country Finland to 20.0% in Lithuania. 

Among young adults (18–24 years), the average % of ROP in the EU was 21.7, whereas in CHANCE partner countries, it ranged from 20.1 in UK to 26.5 in Finland. With respect to adults (of 25–64 years), the lowest % of ROP was found in Finland (10.7%) and the highest was in Lithuania (19.6%), whereas the average value for the EU was 15.1%. In the oldest group (≥65 years), the EU average was 15.9%, whereas Lithuania reported the lowest, and UK had the highest % of ROP at 12.1% and 21.8%, respectively.

**Table 4:** At-risk-of poverty rate (% of ROP, with thousands of people in parentheses) and calculations of the CHANCE total score. Selected data from EUROSTAT 2011 [6] for all countries except Serbia in 2010 [8].

Country	Total Population	Age Category					
		18–24 Years		25–64 Years		≥65 Years	
	% of ROP (Thousands of People)	% of ROP (Thousands of People)	CHANCE Total Score	% of ROP (Thousands of People)	CHANCE Total Score	% of ROP (Thousands of People)	CHANCE Total Score
EU	16.9 (83,472)	21.7 (9192)		15.1 (41,276)		15.9 (13,662)	
Finland	13.7 (725)	26.5 (116)	→3 × 1 = 3	10.7(306)	→1 × 3 = 3	18.9 (174)	→2 × 2 = 4
Italy	19.6 (11,877)	24.9 (1059)	→3 × 1 = 3	17.7 (5936)	→2 × 3 = 6	17.0 (2081)	→1 × 2 = 2
Lithuania	20.0 (647)	26.2 (92)	→3 × 2 = 6	19.6 (340)	→2 × 3 = 6	12.1 (65)	→1 × 1 = 1
Serbia	18.3 (1334)	21.1 (139)	→3 × 1 = 3	17.4 (699)	→2 × 3 = 6	14.8 (183)	→1 × 2 = 2
UK	16.2 (10,018)	20.1 (1153)	→2 × 1 = 2	13.1 (4302)	→1 × 3 = 3	21.8 (2285)	→3 × 2 = 6

Applying the CHANCE total scoring system resulted in the highest scores for ROP among those aged 25–64 years in Italy and Serbia, and for ROP among those aged ≥65 years in Finland and the UK. According to EUROSTAT, Italy has the highest discrepancy related to gender for the risk of poverty, with females at higher risk particularly when <65 years. Therefore, it was decided to discriminate between genders in the Italian population group by considering females only.

The Lithuanian ROP groups aged 18–24 and 25–64 years had equal scores. Because we needed to make a choice between the two, we considered an additional indicator of poverty that was developed in EUROSTAT, namely the so-called “Persistent at-risk-of poverty rate by age group” [6]. For Lithuanian adults it was 8.1%, whereas it was 6% for young adults. Based on this consideration, preference was given to the 25–64 years group in Lithuania.

The final list of study population groups for the expected CHANCE Nutrition Survey comprised adults (25–64 years) for Italy (females only), Lithuania and Serbia, and the elderly (≥65 years) for Finland and the UK.

## 4. Discussion

Although it is generally accepted that earning a low income has a negative impact on the quality of diet, the revision of existing data reported here demonstrates that the information available on nutritional quality and possible malnutrition in economically disadvantaged populations is highly fragmented. The data in the literature came from studies that used different methodologies to assess food consumption and define the quality of the diet. Additionally, the definition of “poverty” is often based on different parameters.

This paper made it clear that information on nutritional dietary habits is especially weak when required for ROP, because the literature data often lack meta-information that could assist in the classification of available data on the basis of income. In relation to the definition and classification of “income” within the study, there were different approaches; the income was expressed as the amount of money available to a household [14,25,31]. The study populations were categorized into two to five income groups [5,12,13,14,15,16,17,18,19,20,22,23,24,26,28,30,32] or other methods were used to identify the low-income groups [11,27,29]. To compare between low- and high-income groups, we had to address comparisons of two extreme groups from some studies and two broad groups from other studies [33]. It is hard to believe that the researchers behind those previous studies were concerned with the harmonization of the key variable (income), and that they were thinking of possible future between-study comparisons as performed in this paper.

It is therefore evident that besides the existing literature, there is a need for a global vision of the primary nutritional problems that are present in low-income people all over Europe. The first step to achieve this global vision is to set a common definition of the risk of poverty based on up-to-date information on income according to demographics, and by using the cut-off point according to the official EU poverty line. Because the gradient of economic inequality may vary greatly between countries, the poverty concept applied in this study is based on the idea that an individual or a family is poor if they have so few resources that they are excluded from the minimum acceptable standard of living in the member state in which they live [11]. This concept fulfils the most central requirement of data quality, which is its suitability for international comparison [34] because it captures the same information across countries. The methodology described here for selecting the ROP groups in different partner countries will assist in obtaining comparable data from the CHANCE nutritional survey, and it could be used in further studies involving not only CHANCE partner countries but also other countries in Europe and around the world.

To uncover evidence of possible malnutrition, a comparison between the current energy and nutrient intake and the recommended intake is required. In published papers in which estimates were compared with dietary reference values, it should be noted that apart from other differences in study methodologies, variations in observed findings between studies might stem from the diversity in the dietary reference values that were applied across studies. Similarly, the studies that were included in this review employed diverse dietary assessment methods. This diversity is another aspect that should be taken into consideration when examining the heterogeneity in results between studies, because it is known that dietary intake measurement errors differ considerably by dietary assessment method [35]. For example, FFQs were the most commonly used dietary instrument in this review, and they are generally designed to rank individuals rather than to assess their absolute intake levels. In addition, a small number of 24HDR replicates with no adjustment for intra-individual variability can yield different estimates in comparison with multiple replicates [36]. The CHANCE survey will be based on an FFQ and two replicate 24HDRs. FFQs are recognized as good instruments to distinguish between subpopulations, although repeated 24-h recalls are often recommended as the most appropriate method of dietary assessment [37]. Recently, it has been asserted that 24HDR administration at least twice is acceptable, and variation can be further reduced by triangulation with other methods, such as FFQ [38].

Studies included in the reported literature search differed in terms of their objectives, methodologies and the ways in which the results were reported, all of which influenced study outcomes. However, the objective of this paper was not to obtain precise estimates of quantitative comparisons between low-income and high-income groups or to estimate the prevalence of intake adequacy, but rather to outline and descriptively summarize evidence about the dietary habits of low-income groups across Europe. Extracting the data as we have done here is the best possible option for answering our research question. In this light, the presence of methodological dissimilarities within the collected dataset can have advantages by increasing the generalizability of the conclusion [39], at least for those foods and nutrients for which data were abundant. 

Despite the different methods for assessing incomes, all the published papers had the same underlying concept, which is the dietary difference between low-income and high-income groups. However, the lack of harmonization between methods in previous papers does not mean that there is no benefit in comparing them with the results that will be obtained with the CHANCE survey. On the contrary, this comparison will allow for the collection of reliable data on dietary habits and on the primary nutritional problems of low-income people in Europe. Based on the results in the literature, all food groups and nutrients classified as critical (see Table 2 and Table 3) will be taken into special consideration in the CHANCE survey.

An added value of this paper is its overview of available evidence and its identification of knowledge gaps relevant to the research question. For example, data on the use of alcohol and intake of vitamins B3, B12, D and E in addition to that of iodine, manganese, phosphorus and selenium were limited (≤two studies). With respect to the setting, not all European countries provided information on dietary habits among low-income groups; however, the repository fairly represents all European regions (Scandinavia, Western Europe, Mediterranean countries and Central and Eastern Europe). Given that the dietary habits of the general population differ across European countries and regions [10], there might be similar tendencies for the dietary patterns of low-income groups. For example, a study on the dietary habits of low-income groups in Poland and Belgium [11] and a study conducted in the Czech Republic, Finland, Poland and Russia [15] both applied uniform methodologies but showed inconsistent findings. Clearly, apart from varying income distributions across countries, traditional dietary patterns could play a role in explaining the observed variations between countries.

Our electronic search strategy employed a comprehensive list of search terms for “income”, and they were previously used in dietary research among low-income groups within the EURRECA project [40,41]. However, the limitation of our search strategy is that we focused on two electronic bibliographic databases and only on articles published in the English language. It is likely that a retrieval of data reported in other languages and from other databases could have added to the existing repository. Nevertheless, at least for the countries involved in the CHANCE project, it can be assumed that the relevant data have been incorporated.

To date, strategies to improve the diet of low-income groups in Europe have addressed changing the physical environment to facilitate lifestyle changes by individuals (access to shops), improvements in nutrition knowledge, the development of local food initiatives *etc.* [42]. Recent work suggests that although these ideas are worthy, most projects reach a small population and are rarely sustained beyond the initial funding [43]. CHANCE is the first EU proposal specifically designed to address the development of nutritionally correct foods and meals targeted to improve the diet of population groups at risk of poverty. The project not only accounts for income, but it also tracks many other determinants that can have an impact on food choices and consequently the food/nutrient intake. For example, foods eaten by people in the lower income groups are higher in energy and lower in micronutrients compared with foods consumed by people in high-income groups, and the levels of physical activity of the low-income groups showed a greater risk of yielding a positive energy balance than the high-income group according to [44].

The CHANCE project investigates not only dietary habits but also physical activity levels and perceived barriers to healthy nutrition in the ROP population, and it integrates all data in addition to cost to achieve a formulation of affordable healthy foods. Implications for the public health policy workforce indicate that future strategies aimed at improving the diet of low-income groups should take behavioral nutrition and the economics of food choice into account [45], which would suggest a promising outcome for this project.

Studying the extent to which reported nutrient intakes are reflected by the food intake results does not give additional explanations to those reported in the results section of this paper because nutrient intake studies (except [13] and [20]) provide information on the consumption of the respective foods, as well. For example, it is evident that low intakes of vitamins A and C, the primary dietary sources of which are fruits and vegetables, are reflected by low intakes of these two food groups. A similar trend holds for low levels of fiber and whole grain foods, whereas low calcium intakes were not confirmed by the intake results for dairy products. It is self-evident that the food and nutrient intakes critical to the nutritional health of ROP groups require quantification and explanation, and this assessment firmly justifies the need for anticipating CHANCE Nutrition Survey.

## 5. Conclusions

The existing evidence for dietary habits of low-income groups in Europe indicates that their diet quality separates them from the more affluent groups. The low-income groups are less likely to consume fresh vegetables and fruits, whole grains, fish and meats, and more likely to consume white bread, starchy foods and processed meat. Additionally, the diet of low-income groups is reported to be low in energy, fiber, vitamins B1, B2, B3, B6, B12 and C, folate, calcium, magnesium, iron, potassium and zinc and to be high in sodium. Unfortunately, the overall reliability of these data is reduced by the different methodologies used in performing the surveys, in addition to differences in defining the risk of poverty threshold.

The aim of the present study is not only to set the stage of existing knowledge, but it is also intended to propose a useful methodology for identifying the primary nutritional concerns of at risk of poverty group across Europe. The proposed scoring system represents novel criteria for defining at-risk-of-poverty groups, not only in the CHANCE participating countries, but all over Europe. The reported summary of existing evidence of food and nutrient consumption patterns among low-income populations in Europe is a pre-requisite for the CHANCE dietary survey. This summary will aim to identify and estimate nutritional criticalities of ROP groups in CHANCE partner countries and contribute to the project objective, *i.e.*, the development of a novel affordable nutritious CHANCE food to promote health and reduce the risk of diet-related diseases among at-risk-of poverty populations. 

Based on these results, all food groups and nutrients present in Table 2 and Table 3 will be analyzed in the CHANCE survey. Moreover, all nutrients available in food composition databases will be considered and in particular those suggested by technologists who are experienced in food reformulations.
